# Spontaneous steinstrasse: A case report and literature review

**DOI:** 10.1016/j.eucr.2025.103268

**Published:** 2025-11-11

**Authors:** Clotaire Alexis Marie Kiemdiba Donega Yaméogo, Moussa Kaboré, Hassami Sawadogo, Brahima Kirakoya, Abdoul-Karim Paré, Tiéoulé Mamadou Traoré, Adama Ouattara, Fasnewindé Aristide Kaboré

**Affiliations:** a Division of Urology, Yalgado Ouédraogo University Teaching Hospital, Ouagadougou, Burkina Faso; b Division of Urology, Ouahigouya Regional Teaching Hospital, Ouahigouya, Burkina Faso; c Department of Urology, Dédougou Regional Hospital, Dédougou, Burkina Faso; d Division of Urology, Souro Sanou University Teaching Hospital, Bobo-Dioulasso, Burkina Faso

**Keywords:** Spontaneous steinstrasse, Renal stones, Ureteric stones, Urinary schistosomiasis

## Abstract

Spontaneous steinstrasse without prior lithotripsy is rare. A 45-year-old male with history of urinary bilharziasis presented with bilateral renal colic. CT scan revealed multiple bilateral renal calculi and a left pelvic ureteral stone cluster measuring 22 × 44 mm. Open surgery retrieved 207 stones from left ureter and 34 from left kidney, requiring ureteral reimplantation. Right ureterolithotomy with resection of bilharzial stenosis was performed. This case is unique for the unprecedented number of stones retrieved and the identification of urinary bilharziasis as underlying etiology, never previously reported. Open surgery remains valuable when endoscopic options are unavailable or complex reconstruction is needed.

## 1 Introduction

Steinstrasse, a German term meaning “street of stones,” describes the radiographic appearance of multiple calculi impacting the ureter.^1^ While commonly a complication of extracorporeal shock wave lithotripsy (ESWL), its spontaneous occurrence is exceptionally rare. Only a handful of cases exist in the literature, often linked to metabolic disorders like renal tubular acidosis.^2^^,^^3^ We report a unique case of spontaneous steinstrasse in a patient with a history of urinary schistosomiasis, notable for the extraordinary number of stones removed and the proposed etiological role of ureteral strictures from past parasitic infection.

## 2 Case presentation

A 45-year-old male attended our clinic with a 4-year history of bilateral renal colic. He had no history of ESWL for renal stones but he had a history of urinary schistosomiasis in childhood for which he received treatment, though detailed records were unavailable. He had no associated comorbidity. His routine physical examination was unremarkable.

**Fig. 1:**
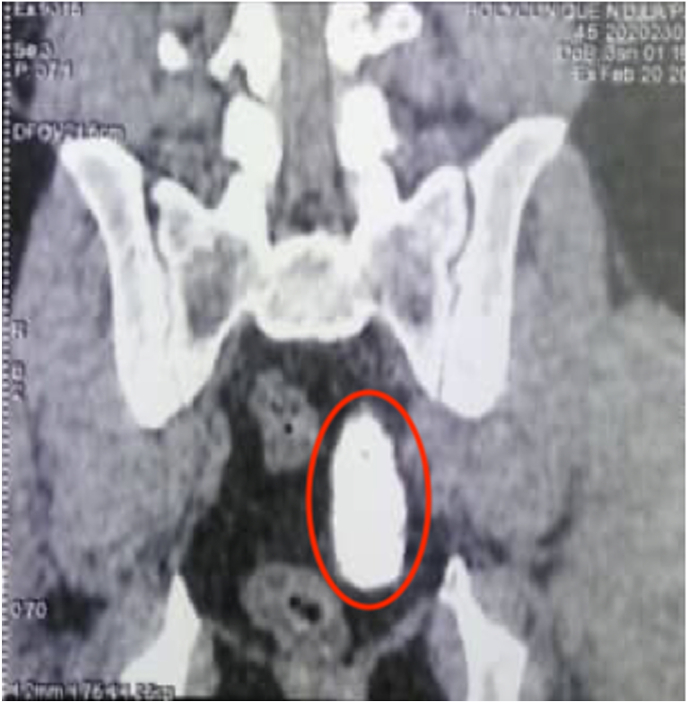
CT scan demonstrating contiguous left distal ureteric calculi circled in red measuring 22mm by 44mm.

**Fig. 2:**
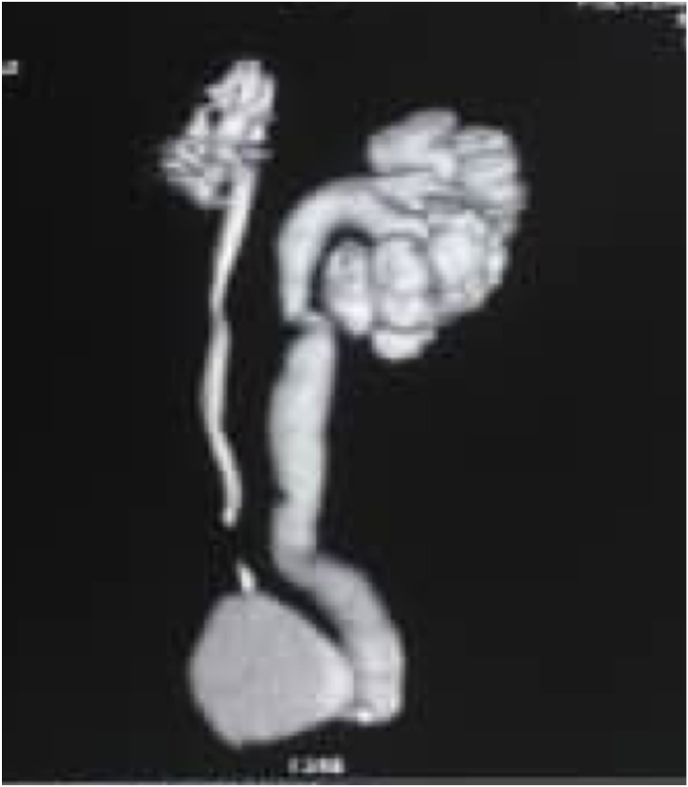
Bilateral uretero-hydronephrosis, more marked on the left side.

Computed tomography scan of the urinary tract revealed multiple bilateral renal stones, an obstructive right pelvic ureteral lithiasis measuring 9 × 11mm, a cluster of obstructive left pelvic ureteral stones measuring 22 × 44mm (Fig. 1). There was a bilateral ureterohydronephrosis, more marked on the left side (Fig. 2). Urine culture confirmed an *E. coli* positive urinary tract infection. His serum creatinine was 1.3mg/dl. The rest of the standard biological tests were normal.

**Fig. 3:**
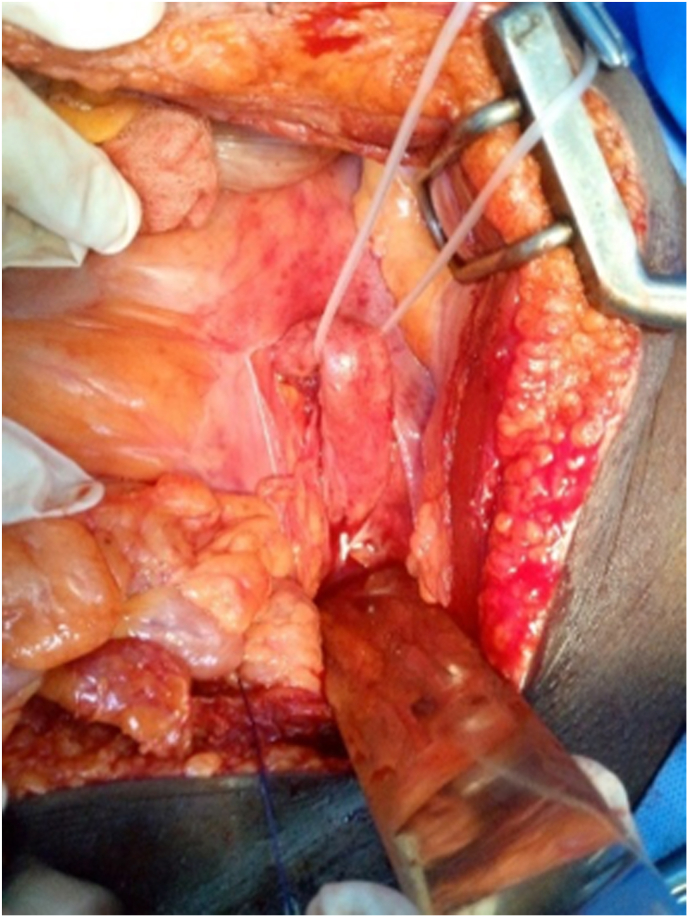
Intraoperative image showing dilatation of left pelvic ureter.

**Fig. 4:**
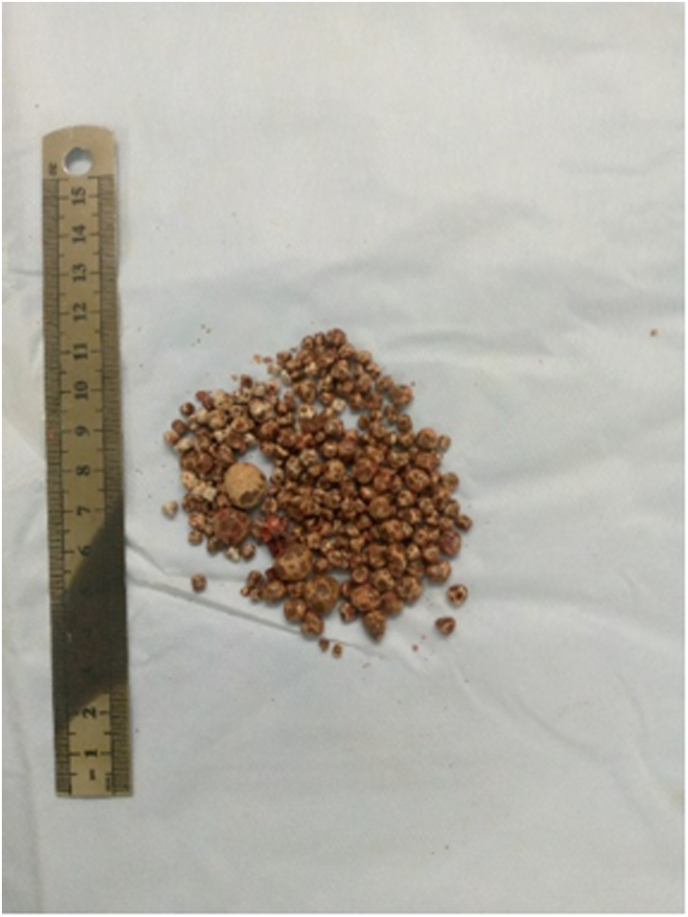
Stones extracted from the left pelvic ureter and left kidney.

**Fig. 5:**
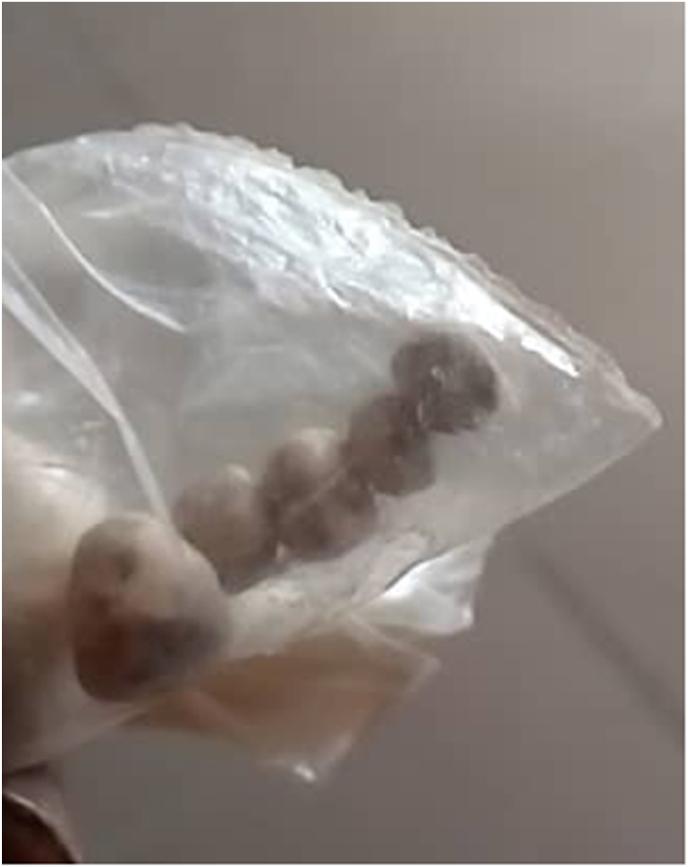
The five stones expelled during micturition.

A midline abdominal incision was performed. Intraoperatively, we noted significant dilatation of the left ureter upstream of a cluster of stones in the pelvic ureter (Fig. 3). After left ureterothomy we removed 207 stones, the largest stone measuring 10mm (Fig. 4). A left nephrotomy was performed through a lower pole incision, and 34 stones were removed. Intraoperative palpation confirmed the removal of the stones, as pyeloscopy was unavailable. We performed a left ureterovesical reimplantation. On the right we performed a pelvic ureterothomy with removal of a stone above of a ureteral stenosis. We then resected the stenotic zone of the right pelvic ureter and performed an end-to-end anastomosis. A double J-stent was inserted into the left and right sides. Double J-stents were removed on postoperative day 21. The postoperative course has been uneventful. One month after surgery, the patient expelled five stones during micturition (Fig. 5). Ultrasound examination of the urinary tract showed no significant residual hydronephrosis and revealed a 10 mm left lower calyceal stone.

## 3 Discussion

Spontaneous steinstrasse is a rare clinical entity. Most reported cases are associated with conditions leading to numerous small renal calculi, such as renal tubular acidosis and medullary nephrocalcinosis.^2^^,^^3^ Parmar et al. reported the largest series of nine cases, with the majority requiring open ureterolithotomy.^4^

Our case is exceptional for two primary reasons. First, the number of stones extracted from the ureter (207) is, to our knowledge, the highest reported from a single ureter in a spontaneous case. Second, we identified urinary schistosomiasis as the likely predisposing factor. Chronic schistosomal infection can cause ureteral strictures, leading to urinary stasis. This stasis facilitates both the formation of calculi in the kidney and their subsequent accumulation in the ureter, creating a spontaneous steinstrasse. The mechanism underlying the formation of numerous small stones rather than a single large calculus may relate to the combination of urinary stasis and a specific metabolic environment conducive to multiple nucleation sites. This specific etiology has not been previously described in this context.

Management of steinstrasse ranges from conservative observation to endoscopic intervention or ESWL.^1^^,^^5^ In this complex scenario, with massive obstruction, bilateral involvement, and the need for ureteral reconstruction (reimplantation and anastomosis), open surgery was the most appropriate and definitive treatment. The subsequent spontaneous passage of stones after surgery suggests that in the absence of anatomical obstructions, conservative management might have been justified, although difficult given the large number of stones in our case.

A limitation of this report is the unavailability of morpho-constitutional stone analysis in our setting, which could have provided further insight into the stone composition. Urinary schistosomiasis creates a predisposition for infection stones (*e.g.*, struvite) due to ureteral strictures causing stasis and recurrent infections. The chronic inflammatory environment and the potential for urease-producing bacterial infections make this the most likely etiology, although it could not be confirmed in our case.

## 4 Conclusion

This case highlights that spontaneous steinstrasse can occur in the absence of metabolic disorders, with urinary schistosomiasis being a potential etiological agent through its complication of ureteral stricture. It also demonstrates that open surgery, though less common in the endoscopic era, remains a crucial and effective option for managing complex urolithiasis, especially in settings with limited resources or when concomitant reconstructive procedures are necessary.

## CRediT authorship contribution statement

**Clotaire Alexis Marie Kiemdiba Donega Yaméogo:** Writing – review & editing, Writing – original draft, Methodology. **Moussa Kaboré:** Writing – review & editing, Writing – original draft, Methodology, Conceptualization. **Hassami Sawadogo:** Writing – review & editing, Writing – original draft, Methodology, Conceptualization. **Brahima Kirakoya:** Writing – review & editing, Writing – original draft. **Abdoul-Karim Paré:** Writing – review & editing, Writing – original draft. **Tiéoulé Mamadou Traoré:** Writing – review & editing, Writing – original draft. **Adama Ouattara:** Writing – review & editing, Writing – original draft. **Fasnewindé Aristide Kaboré:** Writing – review & editing, Writing – original draft.

## Consent

Signed consent was obtained from the patient.

## Conflicts of interest

The authors declare that there are no conflicts of interest regarding the publication of this article.
